# Copper, Cuproptosis, and Neurodegenerative Diseases

**DOI:** 10.3390/ijms26189173

**Published:** 2025-09-19

**Authors:** Giuseppe Genchi, Alessia Catalano, Alessia Carocci, Maria Stefania Sinicropi, Graziantonio Lauria

**Affiliations:** 1 Dipartimento di Farmacia e Scienze della Salute e della Nutrizione, Università della Calabria, Arcavacata di Rende, 87036 Cosenza, Italy; giuseppe.genchi@unical.it (G.G.); s.sinicropi@unical.it (M.S.S.); glauria@unical.it (G.L.); 2 Dipartimento di Farmacia-Scienze del Farmaco, Università degli Studi di Bari “A. Moro”, 70126 Bari, Italy; alessia.carocci@uniba.it

**Keywords:** copper, copper homeostasis, cuproptosis, mitochondria, neurodegenerative diseases, chelating drugs

## Abstract

Copper is a vital micronutrient for animals and plants acting as a crucial cofactor in the synthesis of numerous metabolic enzymes and contributing to mitochondrial respiration, metabolism, oxido-reductive reactions, signal transmission, and oxidative and nitrosative damage. In the cells, copper may exist in the Cu^+^ and Cu^++^ oxidation states and the interconversion between these two states may occur via various redox reactions regulating cellular respiration, energy metabolism, and cell growth. The human body maintains a low level of copper, and copper deficiency or copper excess may adversely affect cellular functions; therefore, regulation of copper levels within a narrow range is important for maintaining metabolic homeostasis. Recent studies identified a new copper-dependent form of cell death called cuproptosis. Cuproptosis occurs due to copper binding to lipoylated enzymes (for instance, pyruvate dehydrogenase and α-ketoglutarate dehydrogenase) in the tricarboxylic acid Krebs cycle. In recent years, extensive studies on copper homeostasis and copper-induced cell death in degenerative disorders, like Menkes, Wilson, Alzheimer, Parkinson’s, Huntington’s diseases, and Amyotrophic Lateral Sclerosis, have discussed the therapeutic potential of targeting cuproptosis. Copper contamination in the environment, which has increased in recent years due to the expansion of agricultural and industrial activities, is associated with a wide range of human health risks. Soil used for the cultivation of grapes has a long history of copper-based fungicide application (the Bordeaux mixture is rich in copper) resulting in copper accumulation at levels capable of causing toxicity in plants that co-inhabit the vineyards. Phytoremediation, which uses plants and biological solutions to remove toxic heavy metals and pesticides and other contaminants from soil and water, is an environmentally friendly and cost-effective technology used for the removal of copper. It requires plants to be tolerant of high levels of copper and capable of accumulating metal copper in plants’ aerial organs and roots. This review aims at highlighting the importance of copper as an essential metal, as well as its involvement in cuproptosis and neurodegenerative diseases.

## 1. Introduction

Copper (Cu) is an essential trace element required by virtually all organisms, from bacteria to humans. Copper plays an important role in mitochondrial respiration, antioxidant defense, cell growth regulation, and synthesis of hormones and neurotransmitters. Copper is also an electron donor or acceptor oscillating between the cuprous reduced (Cu^+^) and the cupric oxidized (Cu^++^) states; it participates in the activity of superoxide dismutase 1 (SOD1) as a cofactor for antioxidant activity. In addition, copper is present in cytochrome C oxidase (COX), terminal enzyme in the electron transport chain for adenosine 5′triphosphate (ATP) synthesis in mitochondria, lysyl oxidase (LOX) for collagen production, tyrosinase for melanin synthesis, ceruloplasmin for iron metabolism, and monoamine oxidase-B (MAO-B) for the oxidative deamination of several amines [1]. The concentration of copper in the adult human body typically ranges from 50 to 120 mg, and it is primarily distributed in the brain, kidneys, liver, and bones. The current recommended daily intake for adults ranges from 0.8 mg/day to 2.4 mg/day [2]. Foods such as sheep liver, nuts, legumes, shellfish, fish, and shrimp contain high levels of copper, while milk, yogurt, and cheese are low in copper. The human body maintains copper levels within the normal range through homeostatic mechanisms of absorption, transport, and excretion. Upon absorption in the gastrointestinal tract, copper reaches the blood bound to ceruloplasmin. In the body, copper cellular transport requires different membrane copper transporters, including copper transporter 1 (CTR1, SLC31A1), divalent metal transporter 1 (DMT1), P-type copper transporting ATPase α (ATP7A), and ATPase β (ATP7B). In addition, various copper chaperones exist, including antioxidant protein 1 (ATOX1), copper chaperon for COX (COX17), and copper chaperon for superoxide dismutase (CCS) [3,4]. Intracellular copper metabolism must be tightly controlled because both excessive and insufficient copper levels are neurotoxic to the cell. In the case of copper overload, excessive copper could translate in an increased production of reactive oxygen species (ROS) through Fenton-like reactions with oxidative damage to proteins, lipids, and nucleic acids, resulting in neuronal dysfunction and cell death. In addition, excessive copper levels in the cells contribute to neuropathogenesis of Wilson, Alzheimer, Parkinson and Huntington diseases, and Amyotrophic Lateral Sclerosis [5]. On the contrary, copper deficiency can hinder growth and immune function, potentially leading to the onset of pathologies like Menkes disease (MD) [6]. In a recent study, Tsvetkov et al. [7] proposed the existence of a new form of copper-induced cell death, namely cuproptosis. The authors underscored that high levels of intracellular copper may induce mitochondrial protein lipoylation, a highly conserved post-translational modification targeting lysine, that occurs in dihydrolipoamide S-acetyltransacylase (DLAT), dihydrolipoamide S-succinyltransacylase (DLST) glycine cleavage system H protein, and dihydrolipoamide branched chain transacylase E2 (DBT)—four enzymes that are associated with the mitochondrial Krebs cycle—resulting in proteotoxic stress and leading to cuproptosis. Among them, DLAT and DLST are subunits of the pyruvate dehydrogenase and α-ketoglutarate dehydrogenase complexes, respectively. The aggregation of these mitochondrial enzymes bound to copper and the loss of the iron–sulfur (Fe-S) cluster induce proteotoxic stress and cell death. Mitochondrial ferredoxin (FDX1) and lipoic acid synthetase (LIAS) are key regulators of copper toxicity, leading to the accumulation of pyruvate and α-ketoglutarate, reducing protein lipoylation and inhibiting Cu-induced cell death. It has been shown that excessive copper accumulation disrupts mitochondrial membranes and their functions by increasing membrane permeability and disrupting membrane potential. Mitochondrial glutathione inhibits cuproptosis, a form of cell death that differs from apoptosis, necrosis, pyroptosis, and ferroptosis, by reducing the thiooctylation process and promoting the oligomerization of DLAT and DLST.

## 2. Chemical Forms and Properties of Copper

Copper (Cu from Latin *cuprum*; atomic number 29, atomic weight 63.55 g/mol, density 8.94 g/cm^3^) belongs to group XI of the periodic table. Copper is a soft, malleable, and ductile metal with very high thermal and electrical conductivities (59.6 × 10^6^ S/m) (Table 1).

Copper is a constituent of various metal alloys, like sterling silver used in jewelry, cupronickel used in marine hardware, and copper–nickel constantan used in thermocouples for temperature measurement. Copper is a native metal that can be employed in a directly usable form. It is present in building construction, usually used for roofing, and it oxidizes to form a green patina of verdigris compound (copper carbonate and sulfate depending upon environmental conditions like sulfur-containing acid rain). A green layer of verdigris can be seen on the Statue of Liberty, in New York City. Since copper reacts slowly, it is often found in its pure native form. About 60% of the copper produced globally is used for electrical wire and cable. In its pure form, copper is an optimum material for wiring because of its electrical conductivity, ductility, low thermal expansion, and corrosion resistance. Copper is also used in plumbing, roofing, integrated circuits (computer chips), coins, electric motors, and cookware. Moreover, copper is used to prepare alloys such as bronze (copper and tin) and brass (mixed with zinc), which are strong enough to make guns and cannons. Copper has been the first metal to be utilized with tin to form bronze since 3500 BC [8]. Pure copper is pinkish-orange, and it acquires a reddish tarnish when exposed to air. Copper reacts slowly with atmospheric oxygen to form a layer of brown-black oxide, which protects the underlying metal from oxidation and corrosion. Common minerals are azurite, malachite, and turquoise with blue or green used as pigments. In nature, copper is present in a variety of minerals, including native copper, chalcopirite, chalcocite, covellite, digenite, azurite, malachite, cuprite, and tenorite. Twenty-nine isotopes of copper are known and among them ^63^Cu and ^65^Cu are stable with ^63^Cu comprising about 69% of total copper. The other isotopes are radioactive; in fact, ^62^Cu is used in ^62^Cu Cu-PTSM [copper pyruvaldehyde bis (N^4^-methylthiosemicarbazone)] complex as a radioactive tracer for positron emission tomography (PET) to evaluate blood flow in the heart, brain, and tumors [9]. In addition, copper compounds are used as bacteriostatic agents, fungicides, and wood preservatives. Copper is also a constituent of tobacco smoke; the tobacco plant absorbs and accumulates heavy metals and also copper into its leaves, which is readily absorbed into a smoker following inhalation [10]. Copper is essential to all living organisms as a trace dietary compound; indeed, it is a constituent of the COX (mitochondrial electron respiratory chain), copper–zinc SOD1 (antioxidant defense), LOX (collagen and elastin crosslinking), and tyrosinase (melanin formation). Found in blood plasma, ceruloplasmin is able to scavenge extracellular radicals like peroxide and superoxide. The copper-containing hemocyanins are proteins, responsible for the binding, transport, and storage of oxygen within the blood of molluscs and crustaceans (blue-blooded *Limulus polyphemus*). Several other functions have been attributed to invertebrate hemocyanin, like enzyme phenoloxidase and hormone transport. The copper ion in hemocyanin switches between Cu^+^ and Cu^++^ states, allowing the protein to change from colorless to blue [11]. While oral copper administration does not cause poisoning, the administration in vapor form can be poisonous. At low concentrations, blue-colored copper salts induce vomiting, while at higher concentrations they cause headaches, migraines, stomach pains, enlarged prostate, liver disorders, and allergic conditions. In normal physiological conditions, the body of a healthy adult contains about 110–120 mg of copper, distributed in the brain, liver, bones, muscles, and blood [12]. For adults, the current recommended daily consumption of copper is about 0.8–2.4 mg/day [13]. Different foods contain various quantities of copper, for example, sheep liver contains 160 mg/kg, lobster contains 36.6 mg/kg, hazelnuts and cashew nuts contain 14.8–22.5 mg/kg, while milk, yogurt, and cheese contain 50–120 µg/L.

## 3. Effects of Copper on Mitochondria

Mitochondria are double membrane organelles, present in all eukariotic cells, originating from bacteria billions of years ago, that merged with proto-eukariotic cells to form an endosymbiotic cellular organism. Mitochondria are structurally similar to bacteria with their own genetic and mitochondrial codes. The mitochondrial genome is composed of a small double-stranded circular DNA molecule. The human mitochondrial chromosome contains 37 genes (16,569 base pairs), including 13 genes that encode proteins for the respiratory chain, and the other 24 genes encode for ribosomal RNA (rRNA) and transfer RNA (tRNA). Nuclear genes encode for all mitochondrial enzymes and protein membrane transporters. The mitochondrial double-membranes (inner and outer phospholipid layers) are separated by an intermembrane space. A mitochondrial outer channel porin (VDAC, Voltage Dependent Anion Channel) is permeable to molecules up to 5000 DA. On the other hand, the inner membrane is permeable to ions and molecules thanks to protein transporters. The inner membrane also serves as an anchor for the components of the electron transport chain (ETC). The mitochondrial respiratory chain plays an essential role in maintaining energy homeostasis through oxidative phosphorylation (OXPHOS), generating energy used to phosphorylate adenosine diphosphate (ADP) to adenosine triphosphate (ATP) in the presence of F_1_F_O_ATP synthase. Mitochondria are also involved in the synthesis of amino acids, lipids, and phospholipids. Moreover, mitochondria are able to modulate cellular differentiation, cell cycle regulation, and cell growth. Several proteins are synthesized in the mitochondrial matrix, like the porphirin ring of the hemeproteins [14]. In addition, mitochondria play an important role in apoptosis (programmed death). This process is usually triggered by the opening of the mitochondrial permeability transition (MPT) pore, that contains porin in the outer mitochondrial membrane, adenine nucleotide carrier in the inner mitochondrial membrane, and cyclophilin D in the matrix [15]. The opening of the MPT pore releases cytochrome C into the cytosol where it interacts with pro-apoptotic members of the B-cell lymphoma 2/Bcl-2-associated protein family. In turn, cytochrome C activates caspase 9, caspase 3, cell shrinkage, membrane blebbing, condensation of chromatin, DNA fragmentation, and cell death. In addition, the opening of the MPT pore allows the influx of solutes into the mitochondrial matrix initiating mitochondrial swelling and rupture [16].

## 4. Absorption and Metabolism of Copper

**Figure 1:**
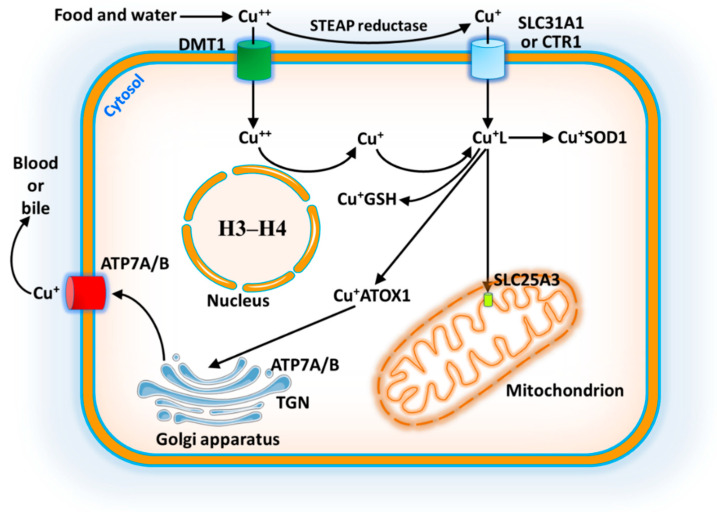
Copper metabolism and trafficking in mammalian cells. The uptake of Cu^+^ via SLC31A1/CTR1 depends on the Cu^++^ reduction to Cu^+^ by six-transmembrane epithelial antigen of prostate (STEAP) family of metalloreductase. In the cytosol of enterocytes, Cu^+^ is bound to a non-proteinaceous ligand, like GSH or L (Cu^+^GSH or Cu^+^L) and then is transferred to SOD1 (Cu^+^SOD1). Copper is delivered to the Golgi lumen thanks to antioxidant ATOX1 and the Golgi Cu pumps ATP7A/B, located in the trans-Golgi network (TGN). Excess copper is pumped out of the cell thanks to ATP7A/B. Copper is also transported to the mitochondrial matrix by the Cu transport protein 3 (SLC25A3). The nuclear histones H3-H4 act as a copper reductase.

Following the digestion of food in the stomach and duodenum, the absorption of copper occurs in the small intestine. The absorbed copper is then transported to the liver, which regulates copper metabolism through the serum carriers ceruloplasmin and albumin. Ceruloplasmin carries about 95% of the copper in plasma, while the remaining 5% is bound to albumin and transcuprein. Cu^++^ cannot be directly absorbed by the enterocytes of the small intestine, because it must first be reduced to Cu^+^ by the six-transmembrane epithelial antigen of the prostate (STEAP) (Figure 1). Extracellular Cu^++^ can also be transported by a divalent metal transporter (DMT1) of enterocytes. Copper is taken up by the small intestine thanks to the carrier copper transport protein 1 (SLC31A1/CTR1), it is exported by the proteins ATP7A/B into the portal vein and then transported to the liver. In the liver, copper is first incorporated into ceruloplasmin and then transported to extrahepatic tissues. Excess copper is secreted into the bile and then excreted in the feces. In the cytosol of enterocytes, copper is bound to a non-proteinaceous ligand (GSH or L) or a metallochaperon, which binds and transfers copper to SOD1. Copper is delivered to the Golgi apparatus thanks to the action of ATOX1 and the Golgi copper pumps ATP7A/B, which are located in the trans-Golgi network (TGN), allowing the synthesis of cuproproteins (lysyl oxidase, ceruloplasim, and tyrosinase) [17]. Recent research by Attar et al. [18] has discovered that nuclear histones H3-H4 act as a copper reductase (Figure 1). Excess copper is pumped out of the cell thanks to the action of ATP7A/B. Copper, bound to non-proteinaceous ligand, is transported into mitochondria, and is translocated to the mitochondrial matrix by the copper carrier transport protein 3 (SLC25A3). Within mitochondria, copper serves as a cofactor of COX and SOD1 (copper–zinc superoxide dismutase) for scavenging free radicals against oxidative damage. In addition, copper, as a cogroup of COX, directly affects proton motive force (PMF) for maintaining mitochondrial respiration, promoting the electron transport chain. In addition to COX and SOD, numerous enzymes have copper as a cofactor, including CuATPases (ATP7A/B), monoamino oxidase (MAO), and COX17. However, the abnormal presence of copper in these organelles disrupts mitochondrial respiration, inducing ROS and mitochondrial dysfunction by enhancing membrane permeability [19,20]. In addition, copper is a rate-limiting nutrient for the growth and proliferation of cancer cells. The oral administration of copper chelator with food has been shown to provide anti-neoplastic and anti-metastatic benefits in human beings [21].

## 5. Copper Trafficking in Mitochondria

**Figure 2:**
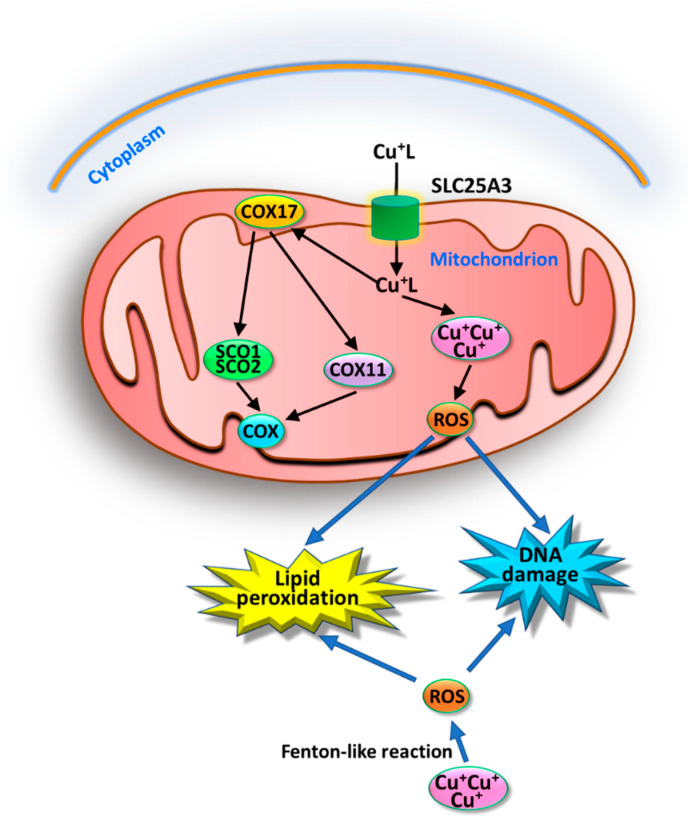
Copper trafficking in mitochondria. Copper bound to a ligand (Cu^+^L) enters mitochondria via outer membrane porin and then SLC25A3 transports it into the matrix. In the matrix copper binds cytochrome C oxidase copper chaperone 17 (COX17) that is a Cu donor to SCO1, SCO2, and COX11 to mediate copper insertion into COX. Excess Cu in mitochondrial matrix, via Fenton-like reaction, induces ROS, lipid peroxidation, and DNA damage.

The non-proteinaceous low molecular weight copper ligand (Cu^+^L) transports copper into mitochondria by means of the solute carrier family 25 member 3 (SLC25A3) [22,23] (Figure 2). Within the mitochondria, copper works with COX to support mitochondrial functions and oxidative phosphorylation (OXPHOS). Cu-cytochrome C oxidase represents the mitochondrial respiratory complex IV, which contains Cu–heme as cofactor for the oxidative phosphorylation. Cytochrome C oxidase copper chaperone 17 (COX17) is a specific copper donor to SCO1, SCO2, and COX11 to mediate copper insertion into COX [24]. COX17 is located in the intermembrane mitochondrial space and is the main copper chaperone that allows the transfer of copper from cytosol to mitochondrial matrix, contributing to the assembly of COX. COX11 is another chaperone that allows the transport of copper from the cytoplasm to COX by means of COX17. Excess copper in mitochondria, thanks to Fenton-like reaction, induces ROS, lipid peroxidation, and DNA damage. In addition, COX17 is a potential target to kill solid or blood cancer [25].

## 6. Cuproptosis Mechanism in Mitochondria

**Figure 3:**
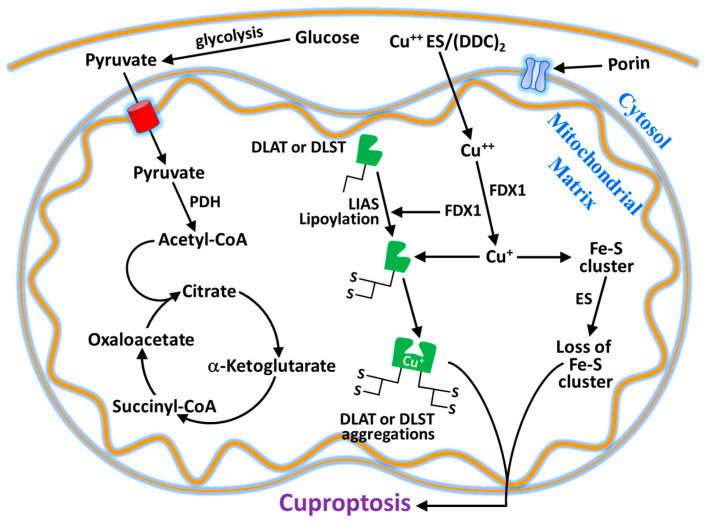
Cuproptosis mechanism. Copper ionophores (elesclomol and diethyldithiocarbamate) capture Cu^++^ to transport it into mitochondria. FDX1 reduces Cu^++^ to Cu^+^ and allows the lipoylation of mitochondrial proteins in the presence of lipoic acid synthase (LIAS). Cu^+^ induces the aggregation of lipoylated proteins and at the same time depletes Fe-S clusters by inducing cuproptosis. The lipoylation is a highly conserved post-translational reaction affecting specific lysine residues in four complexes of Krebs cycle. Among them, dihydrolipoamide S-acetyltransferase (DLAT) and dihydrolipoamide S-succinyltransferase (DLST) are components of pyruvate dehydrogenase and α-ketoglutarate dehydrogenase, which catalyze the conversion of pyruvate to acetyl CoA and α-ketoglutarate to succinyl CoA, respectively. This process of cell death can by mitigated in the presence of copper-chelating agents like tetrathiomolibdate (TTM) or dimercapto succinic acid (DMSA).

It is known that although metallic elements are essential for normal human body functions, excessive levels within cells are deleterious and can lead to cell death. For example, ferroptosis is a form of cell death, that is dependent on iron. In 2022, Tsvetkov and his collaborators [7] showed that excess intracellular copper induces a form of copper-dependent cell death, which they called cuproptosis. The mitochondria are the major target of cell death induced by copper with impaired action of TCA cycle enzymes and oxidative damage of mitochondrial membranes. Cuproptosis depends on excessive level of copper transported into mitochondria by means of the ionophores elesclomol or disulfiram (Table 2), which cannot be rescued by the inhibitor of apoptosis and ferroptosis, except for the action of copper-chelating agents, like tetrathiomolibdate (TTM), D-Penicillamine (DPA), Dimercaptosuccinic acid (DMSA), clioquinol (5-chloro-7-iodo-8-quinoline), and PBT2 (5,7-dichloro-2-[(dimethylamino)methyl]-8-quinoline), which inhibit cuproptosis. Cuproptosis, which affects mitochondria, leads to oxidative damage to the membrane and reduces enzymatic activity in the cell. Copper ionophores (elesclomol or disulfiram) transport Cu^++^ into mitochondria, where Cu^++^ is reduced into Cu^+^ by ferredoxin 1 (FDX1), a mitochondrial matrix reductase (Figure 3). FDX1, a member of the ferredoxin family containing Fe-S cluster, aside from the reduction in Cu^++^ to Cu^+^, allows the lipoylation of mitochondrial proteins by interacting with lipoic acid synthase (LIAS). Proteins of Fe-S clusters are important cofactors for enzymes involved in the electron transport chain. This lipoylation process is a highly conserved post-translational reaction affecting specific lysine residues in four metabolic complexes of TCA cycle, including dihydrolipoamide S-acetyltransferase (DLAT), dihydrolipoamide S-succinyltransferase (DLST), dihydrolipoamide branched chain transacylase E2 (DBT), and glycine cleavage system protein H (GCSH). Among them, DLAT and DLST are important components of pyruvate dehydrogenase (PDH) and α-ketoglutarate dehydrogenase (KDH) complexes of oxidative decarboxylation reaction, where lipoylation is important for their enzymatic function [26]. DLAT and DLST are enzymes of pyruvate dehydrogenase complex and α-ketoglutarate dehydrogenase complex for the conversion of pyruvate to acetyl CoA and α-ketoglutarate to succinyl CoA, respectively, for TCA cycle metabolism (Figure 3). The loss of the FDX1 activity results in the loss of lipoylated protein function, such as the accumulation of pyruvate and α-ketoglutarate. Anyhow, the high presence of Cu^+^ in mitochondria allows the binding of this element to lipoylated proteins, facilitating their aggregation and promoting cuproptosis. Increased levels of Cu^+^ not only promote lipoylated proteins and their aggregation but also weaken the proteins of Fe-S cluster in the presence of FDX1, LIAS, succinate dehydrogenase complex iron–sulfur subunit B (SDHB), and DNA polimerase delta 1 [7]. Taken together, Cu^++^ ionophores (elesclomol or disulfiram), reduction to Cu^+^ thanks to FDX1, lipoylated protein aggregation, and Fe-S loss results in cuproptosis cell death (Figure 3) [7,27].

## 7. Copper and Pathological Conditions

Since both deficiency and accumulation of copper in the human body show negative health effects, it is important to note that the inability to maintain copper balance has been associated with a wide range of pathological states, such as Menkes disease, Wilson disease, and various neurodegenerative diseases (Alzheimer disease, Parkinson disease, Huntington disease, and Amyotrophic Lateral Sclerosis).

### 7.1. Menkes Disease (MD)

Menkes Disease (MD) is a disorder linked to a pathological mutation in the *ATPA7* gene resulting in death during early childhood [28]. MD is estimated to affect 1 in 250,000 live male births, qualifying it as a rare disease. Menkes syndrome is an X-linked recessive disorder caused by mutation in gene coding for ATP7A, leading to copper deficiency. Menkes syndrome is more common in males than in females, like all X-linked recessive pathologies. The mutation in the *ATP7A* gene caused by MD disrupts copper homeostasis in liver, heart, blood, and nerve tissues leading to impaired activities of copper-dependent enzymes, like lysine oxidase, tyrosinase, COX, and dopamine-β-hydroxylase. Studies conducted on infants affected by MD have shown a correlation between copper deficiency in the heart and a congenital cardiovascular abnormality such as the tetralogy of Fallot [29]. The loss of the ATP7A function in the intestine leads to reduced copper efflux in the blood and copper accumulation in the enterocytes. Children diagnosed with MD exhibit severe symptoms, such as intellectual disability, neuronal degeneration, coily and brittle hair, osteoporosis with bone fractures, hypothermia, and vascular abnormalities [30]. In the skin and connective tissues, reduced activity of ATP7A results in decreased function of lipoxygenase which oxidizes lysine and hydroxylisine in elastin and collagen. Children affected by MD present abnormalities in connective tissues including skin laxity, arterial aneurism, and bone fractures. Cu, poorly distributed to cells throughout the body, accumulates in the small intestine and kidneys, while the brain has low levels. There is no cure for this disease; treatment with injections of copper acetate, copper glycinate, or copper histidine are of scarce benefit.

### 7.2. Wilson Disease (WD)

Wilson Disease (WD) (hepatolenticular degeneration) is a rare autosomal recessive disorder of copper metabolism characterized by a mutation in the *ATP7B* gene leading to copper accumulation in the brain, cornea, and liver [31], affecting 1 in 30,000 individuals. In healthy human beings, hepatic copper levels are generally <55 µg/g dry weight. In patients with WD, copper content can be >250 µg/g dry weight [31]. Dystonia, dysarthria, ataxia, and spasticity can be caused by excess copper in the brain. In addition, personality changes, depression, cognitive disturbance, and anxiety can occur as psychological symptoms [32]. Copper deposition in the cornea as a golden–brown ring surrounding the iris (Kayser–Fleischer rings) is a symptom of copper accumulation in the eyes [33]. Patients affected by WD present liver symptoms including fulminant hepatic failure, elevated levels of aspartate and alanine transaminases, and bilirubin in the serum, chronic hepatitis, hepatic cirrhosis, and jaundice [34]. In addition, liver-related symptoms include vomiting, yellowish skin, itchiness, weakness, and swelling in the legs. In WD patients, liver mitochondria are found to have significantly higher copper levels than the nucleus and endoplasmic reticulum as well as changes in the mitochondrial structure, such as separated inner and outer membranes and giant mitochondria [35]. The primary therapeutic strategy for treating WD involves a low-copper diet avoiding the use of copper cookware and the consumption of oral Zn to decrease copper absorption. Copper chelators as cuproptosis reducer are utilized in clinical WD treatment; these drugs bind to copper, facilitating its removal from the body. For the treatment of this disorder, D-Penicillamine, trientine hydrochloride, and tetrathiomolibdate are utilized as chelators (Table 2). Bis-choline tetrathiomolibdate targets intracellular hepatic copper forming a complex with albumin and facilitating copper excretion via bile. Efforts are underway to synthetize new copper chelators. In rats, methanobactin SB2 facilitates the excretion of hepatic excess copper via the biliary pathway [36]. In extreme circumstances, liver transplants can be helpful to those patients for whom other treatments are not effective. Structural brain magnetic resonance imaging (MRI) scansions have shown lesions in midbrain, pons, thalamus, globus pallidus, and cerebellum and cortical atrophy [31].

### 7.3. Alzheimer Disease (AD)

Alzheimer Disease (AD) is the most common neurodegenerative disorder of the central nervous system (CNS) in elderly patients, and it is clinically characterized by memory loss, progressive cognitive decline, and impaired executive functions [37,38]. With an aging worldwide population, the number of AD patients is gradually increasing. At present, it is believed that senile plaques formed by β-amyloid (Aβ) deposition and tau protein hyperphosphorylation are the main pathogenesis of this disorder. Previous reports have shown that Aβ can induce neuroinflammation by activating microglia and also oxidative stress by promoting ROS, which leads to the disruption of mitochondrial membrane potential, lipid peroxidation, DNA damage, and synaptic dysfunction [39]. In addition, studies have shown that dysregulation of copper homeostasis is associated with AD and that the level of copper in the brain of AD patients is significantly higher especially within amyloid plaques [40,41]. Previous studies have shown that elevated levels of copper in both the brain and serum are higher in AD patients compared to healthy individuals of the same age [40]. It has been shown that the presence of 0.1 mg/L of copper in drinking water leads to Aβ deposition in the hippocampus and temporal cortex, impairing learning and memory functions in a mouse model for Alzheimer disease; in addition, trace amounts of copper in drinking water (0.12 ppm) induce Aβ accumulation and learning deficit in a rabbit model of AD [42,43]. The binding of Cu^++^ to Aβ results in the formation of neurotoxic dityrosine-linked Aβ complex [44], which increases Aβ neurotoxicity by promoting the deposition of senile plaques and catalyzing the formation of ROS. Aβ is formed thanks to the cleavage of amyloid precursor protein (APP) in the presence of the enzymes β-secretase and γ-secretase. APP itself has copper-binding sites that allow APP dimerization with enhanced Aβ production and with the formation of neurofibrillary tangles, oxidative stress, inflammatory processes, and ultimately neuronal death [45]. Several copper chelators have been synthesized and tested in mouse models of AD to evaluate the effect of copper-reducing therapy, showing beneficial results like reduced Aβ aggregation, amelioration of neurological symptoms, and antioxidant properties. Cherny and collaborators [46] showed that the chelator clioquinol (Table 2) decreases Aβ deposits and improves both learning and memory capacities in APP transgenic mouse model of AD. The clioquinol derivative PBT2 (Table 2) inhibits Cu-induced Aβ accumulation and demonstrates greater solubility and blood–brain barrier (BBB) permeability. Positive results have been obtained in patients with AD from several phase IIa trials demonstrating that PBT2 could reduce Aβ levels and improve cognitive performance [47]. D-penicillamine reduces serum oxidative stress in AD patients [48]. Cui et al. [49] used nanoparticles to deliver D-penicillamine to the brain because this drug is not able to cross the BBB. Instead, Zhong et al. [50] used chitosan-based hydrogel to transport D-penicillamine to the brain.

### 7.4. Parkinson Disease (PD)

Parkinson disease (PD), which follows AD as the second most frequent neurodegenerative disorder, is characterized by dopamine (Table 2) deficiency in the spinal cord, in the region of the brain known as substantia nigra pars compacta, and by the presence of abnormal aggregates called Lewy bodies. Lewy bodies are enriched in filamentous α-synuclein (small protein of about 20 kDa), which are ubiquitinated before being destroyed [38,51,52,53]. In normal non-pathological conditions, these aggregates are normally destroyed by lysosomes and proteasome complex; however, defects in these cleaning mechanisms, usually in PD, cause further proliferation of these aggregates. Although PD is considered a sporadic disorder, several gene mutations, including α-synuclein, have been identified as causative genes for familial PD. The common clinical manifestations of PD are divided into motor and non-motor symptoms. Motor symptoms include bradykinesia, myotonia, static tremors, and postural balance disturbance, while non-motor symptoms include sleep disturbance, loss of smell, anxiety, depression, and cognitive decline. Postmortem brain analyses of patients with PD have shown increased levels of 4-hydroxyl-2-nonenal (a by-product of lipid peroxidation), DNA and RNA oxidation products, and 8-hydroxy-deoxyguanosine and 8-hydroxy-guanosine [54] in the substantia nigra and striatum, suggesting that excessive levels of ROS and free radicals lead to neuronal damage and death. Dopamine plays an essential function in motor coordination, muscular rigidity, reduced voluntary movement, memory cognition, and depression especially in people over age 60. Levodopa (L-DOPA) (Table 2), the metabolic precursor of dopamine, is used as PD treatment, by restoring the dopamine function in the striatum. In the brain, the enzyme L-DOPA decarboxylase decarboxylates L-DOPA to dopamine. L-DOPA is often administered with carbidopa or benserazide, that are inhibitors of decarboxylation of L-DOPA to dopamine. When L-DOPA is administered over a long time period, undesirable and unpleasant side effects, like dyskinesia, gastrointestinal disorders, chest pain, hives, and weakness begin to appear. At the moment, therapeutic strategies give only symptomatic relief of motor impairment, by supplying L-DOPA and dopamine agonists (pramipexole and bromocriptine) or by inhibiting dopamine breakdown [monoamine oxidase type B (MAO B) inhibitor, β-blocker and adamantine] [55]. However, prolonged treatments with these compounds lead to the development of undesirable harmful reactions. The combination of genetic mutations of α-synuclein, different causes, like aging, environmental factors, oxidative stress injury, and mitochondrial dysfunction are considered promoting factors to PD progression [56]. Copper binds α-synuclein with high affinity in the N-terminal region, promoting its aggregation and also increasing oxidative stress. In 2014, Dikiy and Eliezer [57] discovered that, in the brain, the N-terminal domain of α-synuclein is acetylated. These processes of aggregation are enhanced by reducing the electrostatic repulsion of negative charges [58]. Preclinical studies in PD animal models investigated the clioquinol effect as a therapy, showing also a substantia nigra survival. In PD animal models, the in vivo assessment of the copper complex Cu-ATSM [Cu^++^-diacetylbis(4-methylthiosemicarbazone)] (Table 2) showed an improvement in cognitive performance, restoration of motor functions, and neuroprotection linked to copper release from the complex [59]. Ikawa et al. [60] evaluated the striatal oxidative stress in PD patients using ^62^Cu-ATSM Positron Emission Tomography (PET). To clarify the oxidative stress and mitochondrial dysfunction in living PD patients, the authors have applied PET-^62^Cu-ATSM to functional imaging of oxidative stress due to mitochondrial dysfunction in the striata of PD patients. This PET imaging technique demonstrated that striatal oxidative stress was enhanced in PD patients compared to the controls. Curcumin is emerging as a potential candidate for innovative strategies as a therapy in PD [38].

### 7.5. Huntington Disease (HD)

Huntington Disease (HD) is a rare, progressive autosomal dominant neurological disorder that is eventually fatal [61]. It is characterized by uncontrolled movements, progressive loss of motor and cognitive functions, memory impairment, and neuropsychiatric dysfunctions, like anxiety and depression. This disorder is caused by abnormal amplification of the cytosine–adenine–guanine (CAG) trinucleotide repeat sequence on the short arm of chromosome 4p16.3 within the huntingtin (*HTT*) gene producing the polyglutamine (polyQ) mutant huntingtin protein (mHTT) at the *N*-terminal of this protein [38]. Generally, there are 36–39 glutamine residues in long-chain *N*-terminal polyQ, which produce the cytoplasmic huntingtin protein. In addition, oxidative stress, mitochondrial dysfunction, and transcriptional dysregulation play an important role in HD progression. HTT is involved in many cellular processes including energy metabolism, synaptic activity, neurogenesis and brain development, cell signaling, and vesicle trafficking. Therefore, the aberrant aggregates of this protein lead to the disruption of numerous cellular pathways. It is reported that copper plays a role in HD. In fact, abnormally high levels of copper have been found in the striatum of HD patients and in mouse models of HD compared to controls [62,63]. Fox et al. [62] reported that copper accumulation promotes HTT protein aggregation and interacts with histidine residues at the N-terminal of this protein. The authors reported that copper chelators inhibit the formation of mHTT aggregates, while copper supplementation promotes fibril formation and oligomerization. Furthermore, it is necessary to remember that copper contributes to the progression of HD, because this metal inhibits mitochondrial succinate dehydrogenase (SDH) and lactate dehydrogenase (LDH), both of which are susceptible to Cu-mediated inactivation [64]. In the brain, LDH is important for lactate metabolism providing neurons with energy substrates [65]. These results suggest that copper is involved in the pathogenesis of HD by inhibiting enzymes of lactate metabolism. When considering treatment options, using PBT2 or tetrathiomolibdate mitigates the behavioral abnormalities and the pathology of the R6/2 HD mouse model [66,67]. A study by Lobato et al. [68] on the *Drosophila* model of HD reports that D-penicillamine reduced the synthesis of amyloid-like huntingtin aggregates, thus suggesting a potential therapeutic method to mitigate the toxicity of huntingtin aggregation.

### 7.6. Amyotrophic Lateral Sclerosis (ALS)

Amyotrophic Lateral Sclerosis (ALS) is a rare and progressive paralytic disease characterized by the selective degeneration of motor neurons leading to muscle atrophy, muscular weakness, spasticity, paralysis, and eventually death. ALS is a lethal neurodegenerative disorder, which leads to the progressive loss of both upper and lower motor neurons in the nerve cells, the spinal cord, and brainstem, which control muscle movements and breathing. Upper motor neurons in the brain send signals to the lower motor neurons, which stimulate the muscles involved in walking, talking, eating, and breathing. Two types of ALS are known: sporadic ALS representing 85–90% of cases, which appears without a clear cause, and an autosomal dominant familial form of ALS (FALS) representing 10–15% of cases. About 30–40% of all FALS cases are caused by a defect in the chromosome 9 open reading frame 72 (C9orf72) gene, while 15–20% of familial cases result from a mutation in the *SOD1* gene, which is transmitted to children. The *SOD1* gene is involved in the synthesis of copper–zinc dismutase 1, which catalyzes the dismutation of O_2_^−^ into H_2_O_2_ and oxygen, protecting cells from free radical damage, acting as an antioxidant enzyme. The genetic abnormality leading to neurodegeneration consists of a sequence GGGGCC (guanosine–cytosine) repeated up to 1600 times in ALS patients, while in healthy individuals it is repeated about 20 times [38]. It is known that ALS patients show elevated copper ion levels in the motor cortex (25.1 µg/g in ALS individuals compared to 19.8 µg/g in control individuals), and decreased copper ion levels in the serum (913.21 µg/g in ALS individuals compared to 1020.17 µg/g in controls) [69]. SOD1 is a metalloenzyme that forms stable homodimers by binding copper and zinc ions, in which each monomer binds one copper ion and one zinc ion, respectively, for the activity and stability of the protein [70]; mutations in this enzyme can result in defective copper and zinc binding [71,72]. The SOD1 monomer, after zinc binding and dimerization due to a disulphide bond, takes a copper ion from CCS (copper chaperone superoxide dismutase) and reaches the fully metalated form in this active enzymatic state. About 20% of FALS cases are caused by *SOD1* gene mutation and about 170 SOD1 mutations have been studied. Several well-known mutations, such as G85R, H46R, H80R, 124V, and D125H, have an enzyme characterized by a lower capacity to bind, and therefore, a reduced enzymatic activity [73]. Seetharaman et al. [74] observed that the H80R and D124V variants, present in the zinc-binding area, lead to reduced zinc binding, consequently affecting copper binding. In addition, a mutation of SOD1 in a copper-deficient state leads to misfolded protein aggregates. Therefore, in this situation, copper remains bound to CCS and it is not delivered to other copper-dependent enzymes, thus impairing their activity, e.g., a decrease in mitochondrial respiration [75]. Report by Hottinger et al. [76] showed the beneficial effects and the neuroprotective activity of oral administration of D-Penicillamine, which was able to delay the onset of this disorder and extend the survival in a transgenic mouse model of familial ALS. Tokuda et al. [77] suggested the therapeutic benefit of TTM, which is able to remove copper ion from copper–thiolate clusters, such as SOD1. Moreover, the authors found that TTM exerted therapeutic benefits in the SOD1(G93A) mouse models of ALS.

## 8. Phytoremediation of Copper

Nowadays, industrialization and the extensive use of fertilizers are the major causes of ecosystem contamination. Toxic heavy metals (Pd, Hg, Cd, As, Fe, Ni, Co) and radionuclides, pollution of air, water, and soil are significant environmental problems and even the most invasive and expensive conventional approaches do not provide acceptable solutions. Sewage sludge, which is a good source of plant nutrients, is often used as a soil additive to improve the physical, chemical, and biochemical properties of the soil. The sludge can contain heavy metals, pesticides, detergents, and several other organic materials [78]. Heavy metal contamination and its negative impact on the food chain have been associated with a wide range of significant risks to both human health and other living organisms. While many organic contaminants can be degraded thanks to the microorganisms present in the soil, heavy metals cannot be decomposed through biochemical processes, and continually accumulate in the soil [79], presenting significant risks for human health due to teratogenic, carcinogenic, and mutagenic effects. Soil contamination with heavy metals adversely acts on the natural ecosystem and, through the food chain, adversely affects the CNS and vital organs [80]. Heavy metal contamination of soil includes metal plating, automobile emissions, manure, sewage sludge, industrial wastewater, pesticides, herbicides, fertilizers, and agrochemicals [81]. Chemical, physicochemical and biological methods are known to decontaminate soils polluted with heavy metals; however, these methods are expensive, requiring high amounts of energy or large quantities of chemicals. Phytoremediation is a technology that is non-invasive, cost-effective, highly efficient, and aesthetically pleasing. Metallothioneines, phytochelatings, metallo-enzymes, and many storage proteins are involved in phytoremediation of heavy metals. Phytoremediation uses plants to clean up the environment by extracting, sequestering, accumulating, and depolluting soil, air, and water from the contaminants. High levels of copper in the soil, mainly due to anthropogenic activities, can be dangerous to plants, animals, and human beings. In plants, copper plays a key role in several functions, like photosynthesis, chlorophyll formation, electron transport chain, oxidative stress protection, and metabolism of proteins, carbohydrates, and phospholipids. Although some metals are essential for plants in small concentrations, at higher concentrations they become toxic and cause oxidative stress, forming free radicals. In trace amounts, copper is a transition metal useful for maintaining biochemical and physiological functions. However, excessive copper in soil disturbs cellular homeostasis and reduces plant fitness by inducing photosynthetic impairment. Phytoremediation of heavy metals (including copper) through the use of plants is attracting increasing attention in the scientific literature. Goswami and Das [82] explored the copper phytoremediation potential of *Calandula officinalis* L. following the photosynthesis and antioxidant enzyme activities. The obtained results showed that the *Calandula officinalis* L. had high copper tolerance (up to 400 mg/kg). The authors grew the plants for 21 days in soils with doses ranging from 150 to 400 mg Cu/kg. The pots were placed under natural light and at room temperature. At 150 mg Cu/kg, root and shoot biomass, root lengths, and leaf soluble proteins remained unchanged compared to the control, while chlorophyll and carotenoid content significantly declined with an important increase in lipid peroxidation. At 400 mg Cu/kg concentration, copper accumulation was higher in leaves than in roots. At the 300 mg Cu/kg concentration, copper accumulation was high, with leaf and root accumulation values of 4675 µg/g dry weight and 3995 µg/g dry weight, respectively. At the 200, 250, 300, and 400 mg Cu/kg concentrations, root biomass decreased by 37.3%, 55.2%, 63.4%, and 72.4%, while shoot biomass declined by 16.6%, 56.0%, 66.3%, and 73.6%, respectively, compared to the control. The advantage of using this plant is that it cannot contaminate the food chain, because it is not edible. Zand and Mühling [83] conducted research on the phytoremediation capacity and copper uptake of *Zea mays* L. in copper-contaminated soil. The authors investigated the additions of different quantities of copper (0, 50, 100, 200, and 300 mg Cu/kg) to the soil on the growth parameters of *Zea mays*, copper accumulation in root and shoots, and the phytoremediation of this plant. Copper levels in soil and plant samples were established using atomic absorption spectrophotometry. While the addition of 50 mg Cu/kg stimulated *Zea mays* growth, higher levels of copper showed inhibitory effects on plant growth compared to the control. The addition of copper (50–200 mg/kg) increased copper levels in the roots and shoots of this plant compared to the control treatment. Since the addition of 300 mg Cu/kg to the soil inhibited the plant growth, this treatment was excluded from the experiment. The highest total copper accumulation capacity was 5210.5 µg in the presence of 200 mg Cu/kg. All things considered, the authors found that phytoremediation of copper-contaminated soil by *Zea mays* can be a promising approach in managing copper-contaminated soil. The extensive use of copper-bearing fungicides [Bordeaux mixture, CuSO_4_x5H_2_O-Ca(OH)_2_] in vineyards is responsible for the accumulation of copper in soils. Like other crops, grapevine is susceptible to different diseases including downy mildew caused by *Plasmopara viticola*. Due to the extensive use of copper in viticulture, total copper topsoil concentrations above 100 mg/kg are commonly found [84]. In one case, the soil of a 100-year-old vineyard was discovered to have a total copper concentration above 3000 mg/kg [85]. The phytoremediation technique allows the extraction of copper from vineyard soils that have accumulated copper from the repeated treatment of copper fungicides. Plants like *Plantago lanceolata* L. and *Ricinus communis* L. have high potential to extract and accumulate copper in their tissues at concentrations above 1000 mg/kg, which can be enhanced by the presence of bacteria [86]. Malagoli and collaborators [87] used *Sinapis alba* (an annual plant of Brassicaceae) and *Festuca rubra* cv Merlin (a monocotyledonous grass species) for copper phytoextraction in the vineyard inter-row. The authors first investigated the capacity of plants to accumulate copper in a hydroponic system and then the results were confirmed by growing these plants in pots with polluted soils from six Italian vineyards (five from the Veneto region and the other one from Tuscany). The research from Malagoli et al. showed that *Festuca rubra* cv Merlin accumulated threefold to fourfold copper in the shoots compared to the roots. This plant possesses multi-copper tolerance, likely due to high levels of metallothionein. Tomato plants (*Licopersicon esculentum* L.) [88] were grown for 60 days in triplicate in pots in the presence of different concentrations of copper (250, 500, 700, 1000, and 1500 ppm). At time intervals of 15, 30, 45, and 60 days, plants from each concentration were harvested; the levels of copper, zinc, manganese, and iron were analyzed using atomic absorption spectroscopy. Analysis after 60 days of treatment showed that the aerial parts of tomato plants accumulated more copper than their roots. The results obtained from the researchers indicated that the copper removal from the soil reached 87.7%. In addition, the chlorophyll levels in tomato leaves decreased at low and high concentrations of Cu, while an increase was noted in moderate concentrations.

## 9. Conclusions

The role of copper in cells is multifaceted as a double-edged sword; this metal is an essential cofactor for numerous enzymes, such as Cu/Zn SOD1, COX, and LOX, but, on the other hand, an excess of copper could be toxic, inducing oxidative stress and driving cell death. Recent studies have revealed that a Cu-dependent form of cell death, called cuproptosis, is mediated by the aggregation of lipoylated mitochondrial proteins and by the loss of iron–sulfur cluster proteins, culminating in cell death. Copper mediates the progression of neurodegenerative disorders confirming the alteration in copper quantities in AD, PD, HD, and ALS. The most hereditary diseases associated with copper homeostasis are MD and WD due to copper deficiency and excess, respectively. Currently, copper chelation therapy is usually used to treat diseases related to copper metabolism, like DPA, DMS, trientine, TTM, clioquinol, PBT2, and Cu-ATSM. In addition, current efforts have been focused on the study of new chelating drugs to reduce toxic side effects, increase the ability to cross the BBB and improve patient compliance. However, these treatments can only temporarily alleviate the symptoms of these diseases but cannot prevent or reverse its progression. In addition, the excessive copper exposure can be reduced by avoiding copper-containing supplements, avoiding cooking in copper kettles, avoiding occupational activities with long-term exposure to this metal, and reducing dietary copper intake. In vineyards, *Festuca rubra* and *Sinapis alba* can be periodically mowed from the inter rows and the shoot tissues removed to decrease the copper levels in the soil. The castor bean plant *Ricinus communis* L. and *Plantago lanceolata* L. exhibit copper hyperaccumulating characteristics in vineyard soils contaminated with copper, and copper mining waste, accumulating this metal at concentrations above 1000 mg/kg. *Calandula officinalis* L. showed that high copper tolerance (>400 mg/kg) and copper accumulation was higher in the leaves than in the roots.

## Figures and Tables

**Table 1:** Chemical and physical properties of copper.

Atomic number	29
Atomic weight	63.55 g/mol
Electronic configuration	[Ar] 3d104s1
Melting point	1084.6 °C
Boiling point	2562 °C
Van der Walls radius	140 pm
Density at 20 °C	8.94 g/cm^3^
Heat of fusion	13.26 KJ/mol
Heat of vaporization	300.4 KJ/mol
Molar heat capacity	24.5 J/(mol⋅K)
Pauling electronegativity number	1.90
Electrical conductivity	59.6 × 10^6^ S/m
First ionization energy	745.5 KJ/mol
Second ionization energy	1957.9 KJ/mol
Third ionization energy	3555 KJ/mol
Mohs hardness	3.0
Cristal structure	Face-centered cubic
Oxidation states	−2, −1, 0, +1, +2, +3, +4
Magnetic ordering	Diamagnetic

**Table 2:** Copper-chelating agents and ionophores.

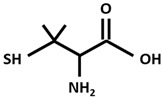	DPA [D-Penicillamine (2-amino-3-mercapto-3-methyl butanoic acid)]
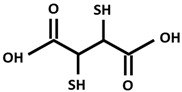	DMSA [Dimercapto succinic acid]
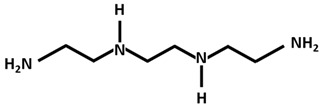	Trientine
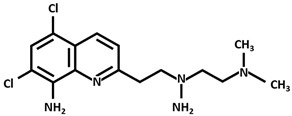	DTMQ20
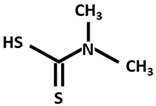	DMDTC [Dimethyldithiocarbamate]
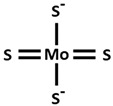	TTM [Tetrathiomolybdate]
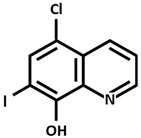	Clioquinol [5-Chloro-7-iodo-8-quinolinol]
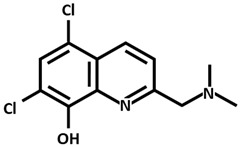	PBT2 [5,7-Dichloro-2-(dimethylamino)methyl-8-quinolinol]
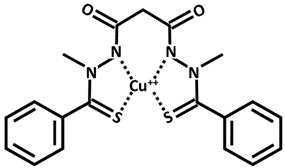	Cu^++^-elesclomol
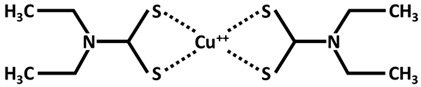	Cu^++^ disulfiram Tetraethyldisulfamdicarbothioamide
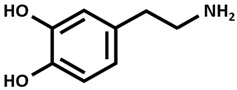	Dopamine
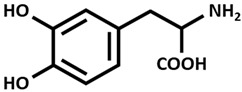	L-DOPA [Levodopa]
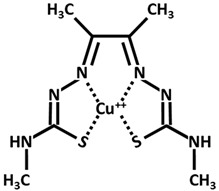	Cu-ATSM [Cu diacetyl bis(*N*^4^-methylthiosemicarbazone)]
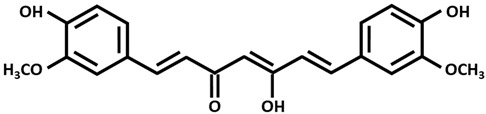	Curcumin (enol form)
